# Involvement of heterologous ubiquitination including linear ubiquitination in Alzheimer’s disease and amyotrophic lateral sclerosis

**DOI:** 10.3389/fmolb.2023.1089213

**Published:** 2023-01-16

**Authors:** Yusuke Sato, Seigo Terawaki, Daisuke Oikawa, Kouhei Shimizu, Yoshinori Okina, Hidefumi Ito, Fuminori Tokunaga

**Affiliations:** ^1^ Center for Research on Green Sustainable Chemistry, Graduate School of Engineering, Tottori University, Tottori, Japan; ^2^ Department of Chemistry and Biotechnology, Graduate School of Engineering, Tottori University, Tottori, Japan; ^3^ Department of Medical Biochemistry, Graduate School of Medicine, Osaka Metropolitan University, Osaka, Japan; ^4^ Department of Molecular and Genetic Medicine, Kawasaki Medical School, Kurashiki, Japan; ^5^ Department of Neurology, Wakayama Medical University, Wakayama, Japan

**Keywords:** ALS, Alzheimer’s disease, cytoplasmic aggregation, LLPS, LUBAC, PROTAC, ubiquitin

## Abstract

In neurodegenerative diseases such as Alzheimer’s disease (AD) and amyotrophic lateral sclerosis (ALS), the progressive accumulation of ubiquitin-positive cytoplasmic inclusions leads to proteinopathy and neurodegeneration. Along with the seven types of Lys-linked ubiquitin chains, the linear ubiquitin chain assembly complex (LUBAC)-mediated Met1-linked linear ubiquitin chain, which activates the canonical NF-κB pathway, is also involved in cytoplasmic inclusions of tau in AD and TAR DNA-binding protein 43 in ALS. Post-translational modifications, including heterologous ubiquitination, affect proteasomal and autophagic degradation, inflammatory responses, and neurodegeneration. Single nucleotide polymorphisms (SNPs) in *SHARPIN* and *RBCK1* (which encodes HOIL-1L), components of LUBAC, were recently identified as genetic risk factors of AD. A structural biological simulation suggested that most of the *SHARPIN* SNPs that cause an amino acid replacement affect the structure and function of SHARPIN. Thus, the aberrant LUBAC activity is related to AD. Protein ubiquitination and ubiquitin-binding proteins, such as ubiquilin 2 and NEMO, facilitate liquid-liquid phase separation (LLPS), and linear ubiquitination seems to promote efficient LLPS. Therefore, the development of therapeutic approaches that target ubiquitination, such as proteolysis-targeting chimeras (PROTACs) and inhibitors of ubiquitin ligases, including LUBAC, is expected to be an additional effective strategy to treat neurodegenerative diseases.

## 1 Introduction

Neurodegenerative diseases, such as Alzheimer’s disease (AD), Parkinson’s disease (PD), amyotrophic lateral sclerosis (ALS), frontotemporal dementia (FTD), Huntington’s disease (HD), and prion diseases, are fatal diseases caused by the progressive loss of structure and function of neurons in the central or peripheral nervous system, and accompanied by protein aggregation and ubiquitin-positive inclusion body formation (Dugger and Dickson 2017; Boland et al., 2018). Importantly, each neurodegenerative disease has typical aggregating proteins, such as amyloid β (Aβ) in AD, tau in AD and FTD, α-synuclein in PD, TAR DNA-binding protein 43 (TDP-43) in ALS and FTD, and mutant huntingtin in HD (Winklhofer et al., 2008). These proteins generally include a low-complexity domain that induces misfolding, oligomerization, liquid-liquid phase separation (LLPS), and aggregation. The aggregated proteins then exhibit proteotoxicity, called proteinopathy, and a microtubule-associated protein, tau-induced pathology, is specifically referred to as tauopathy (Dugger and Dickson 2017). Various post-translational modifications (PTMs), such as phosphorylation, ubiquitination, oxidation, acetylation, SUMOylation, and polyADP-ribosylation (PARylation), regulate the protein homeostasis (proteostasis) of these aggregating proteins. The PTMs also affect the resistance of aggregate proteins toward protein degradation by the ubiquitin-proteasome and/or autophagy-lysosome systems, chronic neuroinflammation, neuronal cell death, and neurodegeneration. Ubiquitin, a 76-residue globular protein, regulates not only proteasomal degradation but also various functions by generating multiple ubiquitin chain linkages. In this review, we focus on the contributions of heterologous ubiquitinations, including the N-terminal Met1 (M1)-linked linear ubiquitination in AD and ALS, and discuss the effects of SNPs on the structure and activity of SHARPIN, which may explain how these SNPs contribute to AD. The ubiquitin system is attractive as a therapeutic target for neurodegenerative diseases. We will therefore focus our discussion on newly developed compounds, such as proteolysis targeting chimeras (PROTACs) and inhibitors for ubiquitin ligases (E3s), which are expected to be potential therapeutic tools to suppress proteinopathies in AD and ALS.

## 2 Ubiquitin code

### 2.1 Ubiquitin code and complex ubiquitination

Protein ubiquitination is one of the major PTMs. The human ubiquitination system comprises two ubiquitin-activating enzymes (E1s), ∼40 ubiquitin-conjugating enzymes (E2s), and >600 E3s, and regulates various cellular functions by producing multiple types of ubiquitin linkages, so-called “ubiquitin code” (Komander and Rape 2012). The C-terminal Gly76 of ubiquitin is reversibly ligated to target proteins or other ubiquitin molecules. Typically, ubiquitin forms polyubiquitin chains *via* seven internal Lys (K) residues, K6, K11, K27, K29, K33, K48, and K63. The most abundant K48-linked ubiquitination predominantly induces proteasomal degradation, whereas the second most K63-linked chain functions signal transduction, DNA repair, and membrane trafficking. Importantly, linear ubiquitin chain assembly complex (LUBAC) is the only E3 that generates the N-terminal M1-linked linear ubiquitin chain through a peptide bond (Kirisako et al., 2006), and is involved in the regulation of the nuclear factor-κB (NF-κB) pathway and apoptosis (Oikawa et al., 2020a). These different ubiquitin chain types are recognized by decoder molecules containing linkage-specific ubiquitin-binding domains (UBDs), and a different signal is activated for each chain (Komander and Rape 2012). Most linkage-specific UBDs, with a few exceptions, bind to Phe4, Ile36, or Ile44-centred hydrophobic patches of ubiquitin but do not recognize around the linkage point, including the C-terminus of ubiquitin (Fennell et al., 2018; Sato 2022). These UBDs contain multiple ubiquitin-binding sites and bind to multiple ubiquitin moieties of the ubiquitin chain with a particular linkage type, thereby increasing the linkage-specific affinity. In addition to mono- and homotypic poly-ubiquitinations, heterologous complex ubiquitinations, with branched and hybrid chains, participate in various cellular functions by multiple cooperating E3s (Ohtake 2022). For example, the K11/K48- and K29/K48-branched chains are reportedly associated with the cell cycle and protein degradation (Meyer and Rape 2014; Leto et al., 2019), whereas the K48/K63-branched and K63/M1-hybrid chains regulate NF-κB signaling (Emmerich et al., 2013; Ohtake et al., 2016). Currently, a large amount (10–20%) of the ubiquitin in polymers is suggested to exist as branched chains (Swatek et al., 2019), and therefore, further studies on the complex architectures of ubiquitin chains are expected. In addition to the ubiquitination of internal Lys residues, non-Lys ubiquitinations, such as thioester-linked ubiquitination of Cys residues, oxyester-linked ubiquitination of Ser/Thr residues, and conjugations of non-protein substrates such as lipopolysaccharide, glycogen, ADP-ribose, and phosphatidylethanolamine, have been identified (Dikic and Schulman, in press; Kelsall 2022; Sakamaki et al., 2022). Therefore, further discoveries of a variety of ubiquitinations that play important pathophysiological roles are anticipated.

### 2.2 Deubiquitinating enzymes (DUBs)

DUBs serve as “erasers” in the ubiquitin code, by removing ubiquitins from substrates, cleaving between ubiquitins, functioning in the biosynthesis of ubiquitin from four genes (*UBB*, *UBC*, *UBA52*, and *UBA80*), recycling ubiquitin prior to proteasomal degradation, editing the ubiquitin linkages, and maintaining the status of the free ubiquitin pool (Komander et al., 2009; Mevissen and Komander 2017). DUBs are also thought to regulate non-Lys ubiquitination, but the details are unknown. There are about 100 human DUBs, which are classified into seven subfamilies: ubiquitin-specific protease (USP), ovarian tumor protease (OTU), ubiquitin C-terminal hydrolase (UCH), Josephin, motif interacting with ubiquitin (MIU)-containing novel DUB (MINDY) (Abdul Rehman et al., 2016), zinc finger with UFM1-specific peptidase domain protein (ZUFSP) (Haahr et al., 2018), and JAB1/MPN/MOV34 metalloenzymes (JAMM/MPN+) (Komander et al., 2009; Mevissen and Komander 2017). The USP, OTU, UCH, Josephin, MINDY, and ZFUBP are cysteine proteases, whereas the JAMM/MPN+ family proteins are zinc metalloproteases. DUBs have different ubiquitin linkage specificities, catalytic activities, and subcellular localizations. Since DUBs are the erasers of the ubiquitin code, they are important in the spatiotemporal regulation of cellular functions, and the failure of the DUB system is associated with many diseases, including neurodegeneration (Komander and Rape 2012; Mevissen and Komander 2017; Bello et al., 2022). Thus, DUBs are crucial targets for drug discovery. Although multiple DUBs are cooperatively involved in the regulation of complex ubiquitin chains, the details remain unknown.

### 2.3 LUBAC-mediated linear ubiquitination and its regulators

**FIGURE 1:**
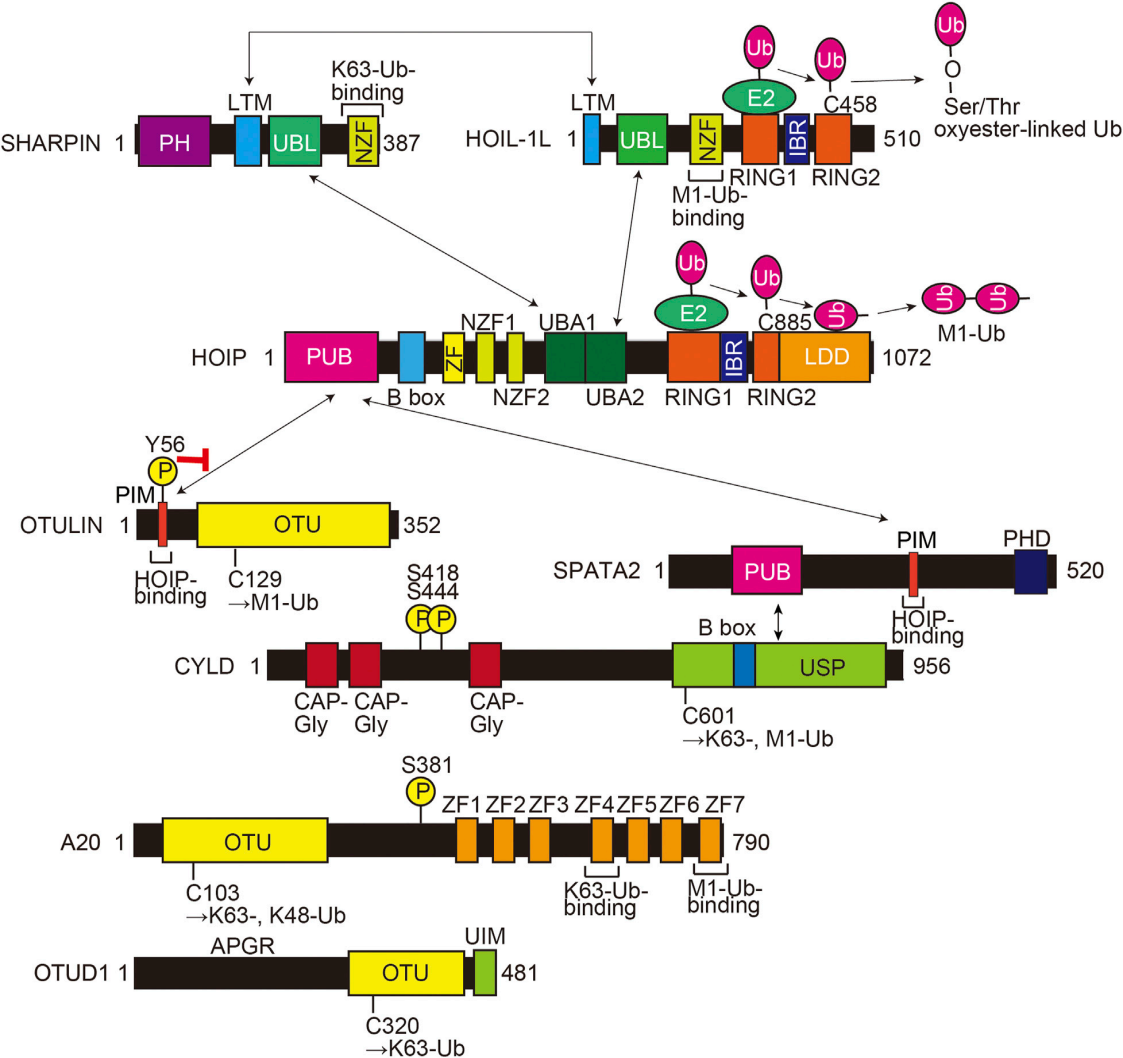
Domain structures and interactions of LUBAC subunits (HOIL-1L, HOIP, and SHARPIN) and LUBAC-related DUBs (OTULIN, CYLD-SPATA2, A20, and OTUD1). The interaction sites are shown by arrows, and a red inhibitory arrow indicates suppressed HOIP-binding by phosphorylation of Tyr56 in OTULIN. The E3 catalytic mechanisms of HOIL-1L and HOIP are also indicated. The active center and ubiquitin chain specificity of each DUB are shown. PH, Pleckstrin-homology; LTM, LUBAC-tethering motif; UBL, ubiquitin-like; NZF, Npl4-type zinc finger; RING, really interesting new gene; IBR, in-between RING; PUB, PNGase/UBA or UBX; B box, B-box-type zinc finger domain; ZF, zinc finger; UBA, ubiquitin-associated; LDD, linear ubiquitin chain determining domain; PIM, PUB domain-interacting motif; OTU, ovarian tumor protease; PHD, plant homeodomain; CAP-Gly, cytoskeleton-associated protein Gly-rich domain; USP, ubiquitin-specific protease; APGR, Ala-, Pro-, and Gly-rich region; UIM, ubiquitin-interacting motif; Ub, ubiquitin; encircled P, phosphorylation sites.

LUBAC is an E3 complex composed of the SHARPIN, HOIL-1L (also known as RBCK1), and HOIP (RNF31) (Figure 1) (Tokunaga and Ikeda 2022). The ubiquitin-like (UBL) domains in SHARPIN and HOIL-1L bind to the ubiquitin-associated (UBA)1 and UBA2 domains, respectively, in HOIP (Fujita et al., 2018). The interaction between LUBAC-tethering motifs (LTMs) in SHARPIN and HOIL-1L further stabilizes the complex. HOIL-1L and HOIP are classified as RING-IBR-RING (RBR)-type E3s, which catalyze polyubiquitination through a RING-HECT-hybrid reaction (Wenzel et al., 2011; Dove et al., 2016). During the linear ubiquitination, the RING1 domain in HOIP binds a ubiquitin-charged E2, and then, the donor ubiquitin is transferred to the active Cys885 in the RING2 domain of HOIP. The donor ubiquitin is finally conjugated to an acceptor ubiquitin, which is held in the linear ubiquitin chain determining domain (LDD), and generates an M1-linked ubiquitin chain (Figure 1) (Stieglitz et al., 2012b; Smit et al., 2012; Stieglitz et al., 2013; Lechtenberg et al., 2016). In contrast, HOIL-1L uniquely catalyzes the oxyester-linked ubiquitination of Ser/Thr residues through the active Cys458 (Kelsall et al., 2019), and SHARPIN is a non-enzymatic regulatory subunit of LUBAC (Gerlach et al., 2011; Ikeda et al., 2011; Tokunaga et al., 2011). Upon stimulation with inflammatory cytokines such as TNF-α, LUBAC conjugates an M1-polyubiquitin chain onto NF-κB-essential modulator (NEMO), receptor-interacting Ser/Thr kinase 1 (RIPK1), and other proteins. The linear ubiquitination of these proteins activates the canonical IκB kinase (IKK) complex, composed of the kinase subunits IKKα and IKKβ, and a regulatory subunit of NEMO. Since NEMO includes a ubiquitin binding in ABIN and NEMO (UBAN) domain, which specifically binds the M1-ubiquitin chain (Rahighi et al., 2009), LUBAC and the resulting M1-ubiquitin chain function as a scaffold and recruit multiple IKK complexes to activate the NF-κB pathway.

HOIP has a PNGase/UBA or UBX (PUB) domain at the N-terminal portion, which plays an important role to recruit DUBs such as OTULIN (Schaeffer et al., 2014) and the CYLD-SPATA2 complex (Elliott et al., 2016; Kupka et al., 2016; Schlicher et al., 2016; Wagner et al., 2016) (Figure 1). OTULIN is an OTU-family DUB, and that binds to the PUB domain of HOIP through the PUB domain-interacting motif (PIM), and the phosphorylation of Tyr56 in the PIM suppressed binding to HOIP (Elliott et al., 2014; Schaeffer et al., 2014; Takiuchi et al., 2014). OTULIN exclusively hydrolyzes the M1-linked ubiquitin chain and regulates the LUBAC-mediated innate immune responses (Fiil et al., 2013; Keusekotten et al., 2013; Rivkin et al., 2013). In contrast, CYLD, a member of the USP family, is a bi-functional DUB that hydrolyzes K63- and M1-linked ubiquitin chains, and downregulates the NF-κB activation pathway (Regamey et al., 2003; Trompouki et al., 2003; Sato et al., 2015). Interestingly, the CYLD-SPATA2 complex binds to the PUB domain of HOIP through the PIM in SPATA2 (Figure 1) (Elliott et al., 2016; Kupka et al., 2016; Schlicher et al., 2016; Wagner et al., 2016). We reported that A20 and OTUD1, OTU-family DUBs, also downregulate LUBAC-induced NF-κB activation (Figure 1). Although A20 cleaves K63- and K48-ubiquitin chains, but not the M1-ubiquitin chain, A20 downregulates LUBAC-induced NF-κB activation by specifically binding to the M1-ubiquitin chain through the zinc finger 7 (ZF7) domain (Tokunaga et al., 2012). In contrast, OTUD1 extensively removes K63-ubiquitin chains in LUBAC and TNF-α receptor complex I, thus regulating the canonical NF-κB, KEAP1-mediated antioxidant response, and reactive oxygen species (ROS)-associated cell death pathways (Oikawa et al., 2022). Collectively, these findings indicate that LUBAC and its related DUBs build and scrap linear ubiquitin chains, and thus participate in various pathophysiological phenomena.

## 3 Heterologous ubiquitin chains in neurodegenerative disease inclusions

### 3.1 AD and heterologous ubiquitination

Sporadic AD, the most common cause of dementia, involves the heterogenous interactions of genetic and environmental risk factors, whereas familial AD is a rare autosomal dominant disease caused by genetic mutations in the amyloid precursor protein and presenilin genes, which function in Aβ metabolism (Scheltens et al., 2021). The intracellular neurofibrillary tangles (NFTs), composed of hyperphosphorylated tau, and the extracellular Aβ plaques are the main pathological hallmarks of AD. The ubiquitin-proteasome system plays an essential role in the pathogenesis and progression of AD, and the involvement of multiple ubiquitin chains in AD has been reported. Cripps et al. showed that K48-linked polyubiquitination is the primary form in paired helical filaments of hyperphosphorylated tau, whereas K6- and K11-ubiquitin chains are included as minor portions (Cripps et al., 2006). Furthermore, Dammer et al. identified that, in comparison to normal brains, the K11-, K48-, and K63-ubiquitin chains, but not the K29-chain, are increased in AD specimens, and that while the K11- and K48-ubiquitinations are suggested to be correlated with proteasomal degradation, the enhanced K63-ubiquitination regulates autophagy-lysosomal degradation (Dammer et al., 2011). Recently, Puangmalai et al. reported that K63-linked ubiquitinated, but not K48 ubiquitinated, soluble tau oligomers accumulate in AD brains, and are associated with enhanced seeding activity and pathological propagation (Puangmalai et al., 2022). These results suggested that multiple E3s are involved in AD-associated ubiquitination. In AD patients, various E3s, such as NEDD4-1, MARCH8, RNF192, Itch, and TRAF6, are reportedly upregulated and/or activated, whereas TTC3, Ube3A, CHIP, HRD1, and Parkin are downregulated (Potjewyd and Axtman 2021). We reported that M1- and K63-ubiquitins are colocalized with thick bundles of tau NFTs from AD patients, while K48-ubiquitin is present in both tiny and thick inclusions (Nakayama et al., 2019). Therefore, LUBAC and its linear ubiquitination activity seem to be involved in the tauopathy and progression of AD. Furthermore, DUBs such as UCHL1, USP10, and USP11, as well as the E2 enzyme of E2-25K/HIP-2, and a frameshift ubiquitin mutant with 20 extra amino acid residues at its C-terminus (UBB^+1^), are reportedly involved in AD (Upadhya and Hegde 2007; Wei et al., 2022; Yan et al., 2022). Women are 1.7 times more susceptible to AD than men. Interestingly, Yan et al. recently reported that X-linked USP11 removes K48- and K63-ubiquitin chains bound to K281 of tau, which increases the acetylations of K281 and K274, resulting in the enhanced aggregation in women (Yan et al., 2022). Thus, the DUB function of USP11 is correlated with sex differences in AD onset. These findings suggest that ubiquitination and deubiquitination are deeply associated with the onset of AD.

### 3.2 SNPs in *SHARPIN* and *HOIL-1L* are genetic risk factors for AD

**FIGURE 2:**
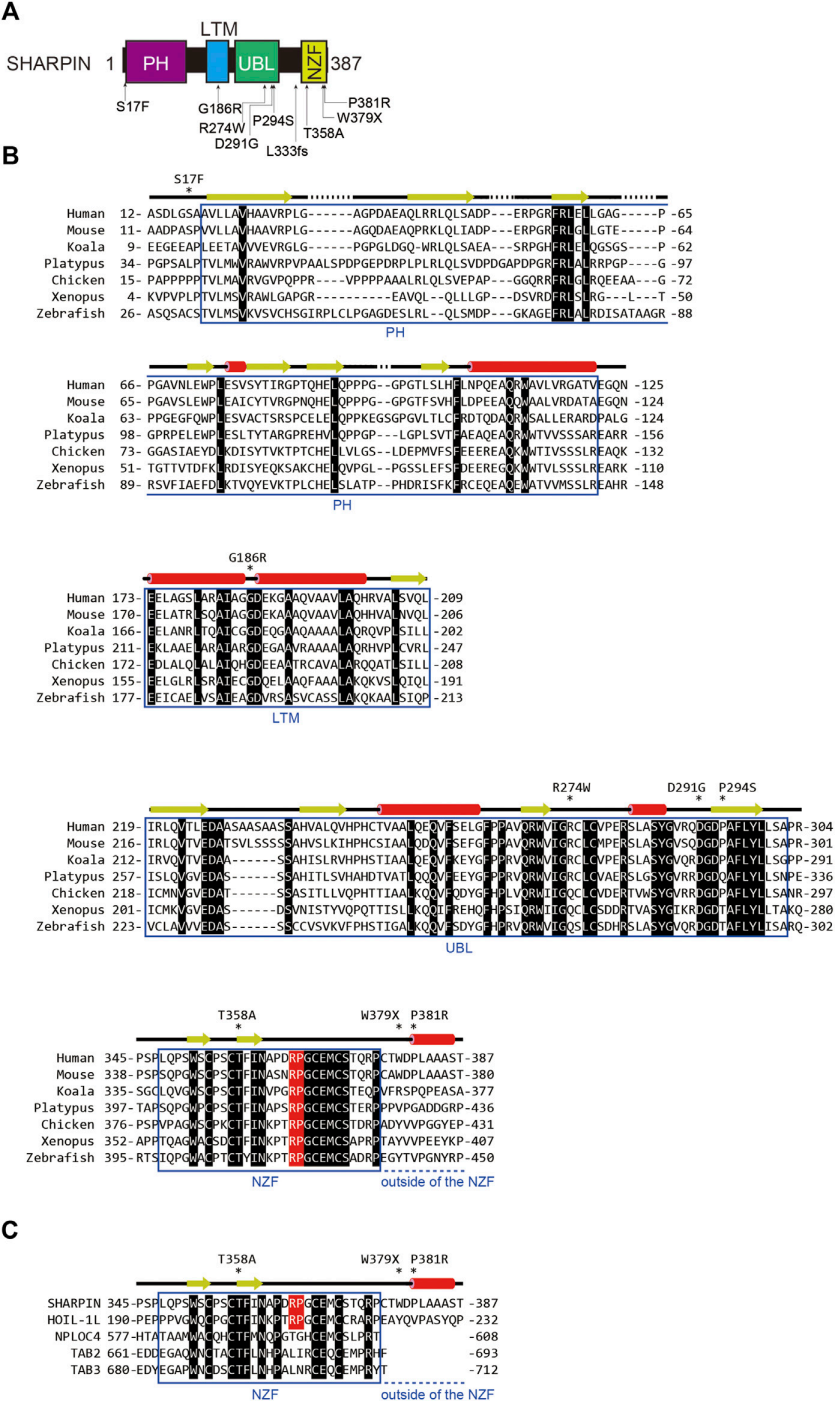
AD-associated SNP sites and the amino acid alignment of SHARPINs from various species. **(A)** AD-associated SNPs are mapped on the domain structure of SHARPIN. **(B)** The amino acid sequences of SHARPINs from various species are aligned. Residues conserved in all species are shown by white letters on a black background. The Arg-Pro sequences conserved between the NZF domains of HOIL-1L and SHARPIN are highlighted by a red background. Arrows: β-sheet; red cylinders: α-helix. **(C)** The NZF domain of SHARPIN showed the highest similarity with that of HOIL-1L. The amino acid sequences of various ubiquitin-binding NZF domains are aligned and structurally characterized as in **(B)**.

Over 70 loci have been identified as AD-associated genetic risk factors, with *APOE* and *TREM2* as major factors. Recent genome wide association study (GWAS) analyses showed that single nucleotide polymorphisms (SNPs) in *SHARPIN* and *RBCK1* (which encodes HOIL-1L) are genetic risk factors for late-onset AD (Table 1; Figure 2A), indicating that LUBAC is correlated with AD. Importantly, the genetic deficiency of *Sharpin* in mice (*cpdm* mice) causes early-onset severe dermatitis (Gijbels et al., 1995), and mutations in human *RBCK1* are known to cause polyglucosan body myopathy type 1 (PGBM1), with or without immunodeficiency (OMIM ID; 610924) (Boisson et al., 2012). Most of the AD-associated *SHARPIN* variants cause amino acid replacements, whereas the AD-associated *RBCK1* SNP (rs1358782) is an intron variant (Bellenguez et al., 2022). Among them, the G186R and R274W variants of SHARPIN reportedly suppressed TNF-α-mediated NF-κB activation and generated aberrant granular clumps in HEK293T cells (Asanomi et al., 2019; Asanomi et al., 2022). Moreover, the R274W variant of SHARPIN shows a weaker interaction with HOIP (Park et al., 2021). As an etiology of AD, SHARPIN reportedly regulates Aβ phagocytosis, inflammation, and cell death in macrophages by linking to the NLRP3 inflammasome in response to Aβ (Krishnan et al., 2020). The siRNA-mediated knockdown of *SHARPIN* ameliorates Aβ phagocytosis, M1 polarization of macrophages, neuroinflammation, and oxidative stress.

**TABLE 1:** AD-associated variants of *SHARPIN* and *HOIL-1L*.

Gene	dbSNP ID	Chr:Position	Change	Domain	Predicted functional changes	Reported functional changes	References
*SHARPIN*	rs34173062	8:145158607	p.Ser17Phe (G/A)	Near PH domain	Not involved in homodimerization of SHARPIN and little effect on LUBAC activity	NA	Soheili-Nezhad et al. (2020); de Rojas et al. (2021); Bellenguez et al. (2022)
	rs572750141	8:145154709	p.Gly186Arg (C/T)	LTM domain	Destabilizing LUBAC by reduced interaction with LTM in HOIL-1L	Decreased NF-κB activity and granular accumulation	Asanomi et al. (2019)
	rs77359862	8:145154282	p.Arg274Trp (G/A)	UBL domain	Reduced interaction with UBA in HOIP	Decreased NF-κB activity and formation of cytoplasmic clumping	Park et al. (2021); Asanomi et al. (2022)
						Reduced interaction with HOIP	
	rs1378764618	8:145154230	p.Asp291Gly (T/C)	UBL domain	Loss of hydrogen bond with Q481 in HOIP	NA	Asanomi et al. (2022)
	rs34674752	8: 145154222	p.Pro294Ser (G/A)	UBL domain	Loss of hydrogen bond with M484 in HOIP and destabilizing SHARPIN UBL fold	NA	de Rojas et al. (2021)
	NA	8:145154035	p.Leu333fs (C/-)	Between UBL and NZF domains	Loss of ubiquitin binding	NA	Asanomi et al. (2022)
	rs201818510	8:145153873	p.Thr358Ala (T/C)	NZF domain	Reduced affinity for M1-linked chain	NA	Asanomi et al. (2022)
	NA	8:145153808	p.Trp379* (C/T)	Outside of the NZF domain	Little importance	NA	Asanomi et al. (2022)
	NA	8:145153803	p.Pro381Arg (G/C)	Outside of the NZF domain	Little importance	NA	Asanomi et al. (2022)
*RBCK1 (HOIL-1L)*	rs1358782	20:413334	intron variant (G/A)	NA	NA	NA	Bellenguez et al. (2022)

NA, not assigned.

**FIGURE 3:**
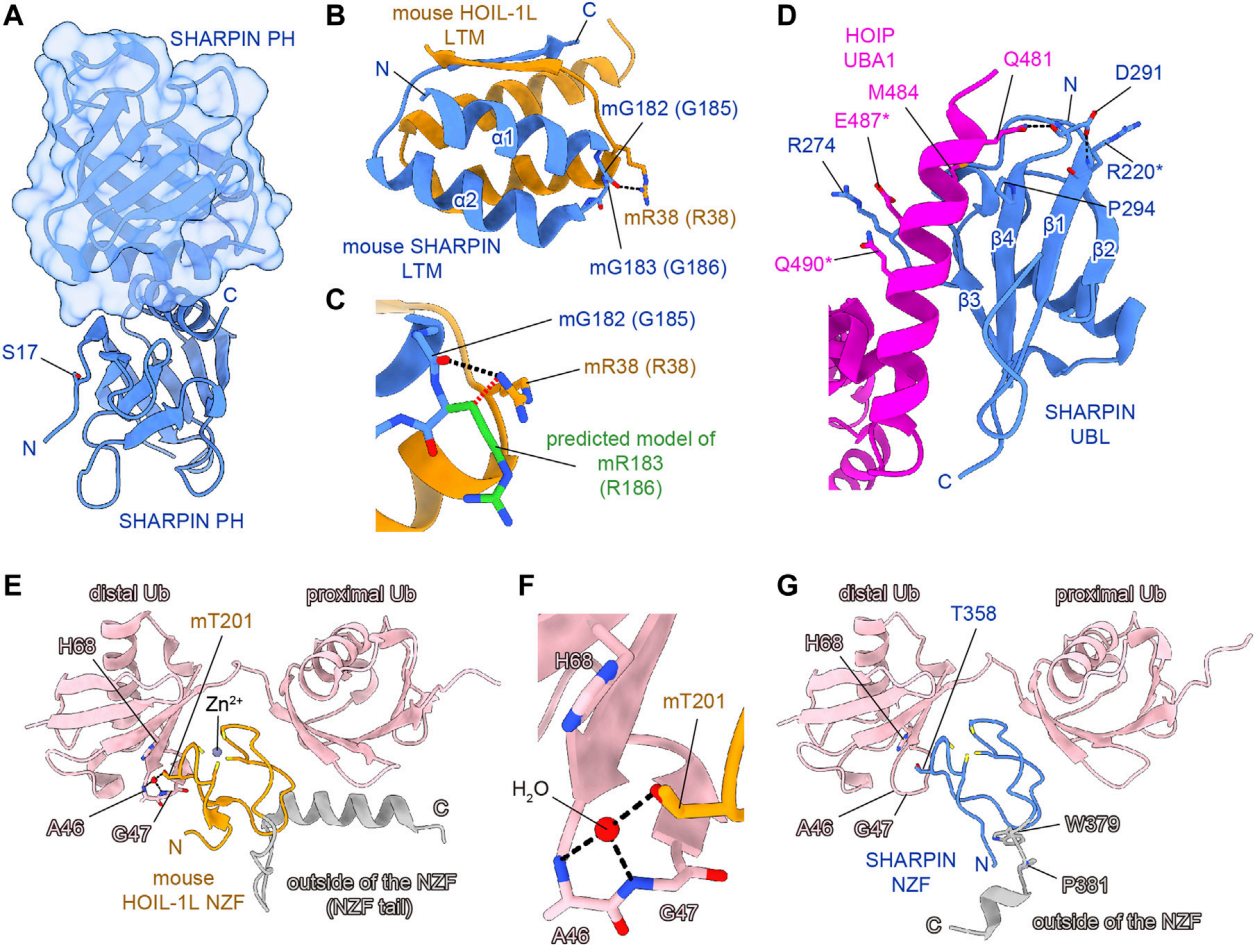
Structural simulations of AD-associated SHARPIN SNP sites. SHARPIN, HOIL-1L, HOIP, and M1-linked diubiquitin are colored blue, orange, purple, and pink, respectively. Hydrogen bonds are shown as black dashed lines. **(A)** Crystal structure of the human SHARPIN PH homodimer (PDB 4EMO). One protomer is shown as a cartoon model, and the other is shown as a translucent surface and cartoon model. **(B)** Close-up view of the interface between the SHARPIN LTM and HOIP LTM in the crystal structure of the hetero-trimeric core of mouse LUBAC (PDB 5Y3T). Numbers in parentheses indicate the corresponding human protein residues. **(C)** Structural simulation of the G183R mutation of mouse SHARPIN (equivalent to G186R mutation of human SHARPIN). The predicted model of mR183 is colored green. Rotamers of mR183 are selected to be in the opposite orientation to the mR38 of mouse HOIL-1L. The steric hindrance between the C_β_ of SHARPIN mR183 and HOIL-1L mR38 is indicated by the red dashed line. **(D)** Close-up view of the interface between the SHARPIN UBL and the HOIP UBA1 in the crystal structure of the human SHARPIN UBL and HOIP UBA1-UBA2 complex (PDB 5X0W). Asterisks indicate residues with weak electron density and no side chains, according to the PDB model file. **(E)** Crystal structure of the mouse HOIL-1L NZF and M1-Ub_2_ complex (PDB 3B08). **(F)** Close-up view around the mT201 of mouse HOIL-1L NZF in **(E)**. **(G)** Structure of the human SHARPIN NZF and M1-Ub_2_ complex predicted by AlphaFold2. Since AlphaFold2 currently cannot predict a non-protein structure, the zinc ion and water molecules are not shown. Regions outside of the NZF are colored gray in **(E,G)**.

To clarify the effects of AD-associated SNPs in *SHARPIN*, we will start from a structural biology point of view. The amino acid sequence alignment of SHARPINs from various species showed that some SNPs occur at evolutionarily conserved amino acids and/or in the functional domains (Figure 2B). For instance, the S17F mutation is located near the N-terminal PH domain of SHARPIN (Figures 2A, B) (Stieglitz et al., 2012a). Although there is no sequence homology, many proteins use the PH superfold as a lipid- or protein-binding domain (Blomberg et al., 1999). Residues 20–121 of SHARPIN form a stable PH superfold, and the C-terminal α-helices (residues 105–121) of the two molecules of the SHARPIN PH domain interact with each other to form a homodimer, with a *K*_d_ value of 88–160 μM (Stieglitz et al., 2012a). In contrast, residues 1–19 of SHARPIN form a flexible loop, and S17 is either disordered or exposed to the solvent in the reported crystal structure (Figure 3A). Therefore, at least in the crystal structure, S17 is not involved in homodimerization. In addition, S17 is conserved in human and mouse, but not other species, suggesting that S17 is not essential for the formation of the PH fold and its homodimerization (Figure 2B). Current data suggest that the SHARPIN PH domain does not contribute to the NF-κB activation by LUBAC, and the function of the PH domain remains enigmatic (Tokunaga et al., 2011). Future functional analyses of the PH domain will be necessary to elucidate the impact of the S17F mutation on SHARPIN.

Next, the LTM domain (residues 173–209) and the UBL domain (residues 219–302) of SHARPIN bind the UBL domain of HOIL-1L and the UBA1 domain of HOIP, respectively (Fujita et al., 2018). These interactions are essential for the LUBAC complex formation and the activation of the RBR domain of HOIP. Therefore, the LTM and UBL domains of SHARPIN are necessary for the catalytic activity of LUBAC. The G186R mutation of *SHARPIN* is located in the LTM domain (Figures 2A, B). The Gly residue at this position in SHARPIN is highly conserved among species, indicating its importance (Figure 2B). The structure of the human SHARPIN LTM domain has not been determined, but the trimeric core structure of the mouse LUBAC (SHARPIN LTM-UBL, HOIL-1L LTM-UBL, and HOIP UBA1-UBA2) was reported (Fujita et al., 2018). G183 of mouse SHARPIN (mG183, equivalent to G186 of human SHARPIN, with the prefix ‘m’ indicating an amino acid residue of mouse SHARPIN) is located in the turn region that connects the α1 and α2 helices with important Phi and Psi angles that typically can be accommodated by glycine but not by other residues, hence the mG183R mutation predicted to significantly affect the structure and function of the SHARPIN LTM domain (Figure 3B). Furthermore, R38 of mouse HOIL-1L (mR38, equivalent to R38 of human HOIL-1L) forms a hydrogen bond with the main-chain CO group of G182 in mouse SHARPIN (mG182, equivalent to G185 of human SHARPIN), while the mG183R mutation of SHARPIN would cause steric hindrance and electrostatic repulsion with HOIL-1L mR38 (Figure 3C). As the C_β_ atom of SHARPIN mR183 would be located 2.1 Å from the N_η_ of HOIL-1L mR38, all rotamers of mR183 are predicted to present severe clash with mR38. Due to these effects, the G186R mutation of SHARPIN would impair the formation of the LUBAC complex. Since the G186R mutation of SHARPIN reportedly resulted in aberrant intracellular localization and attenuated NF-κB activation (Asanomi et al., 2019; Asanomi et al., 2022), the G186R mutation may cause dissociation of the LUBAC complex, and then the destabilized SHARPIN could form aggregates in the cell.

The R274W, D291G, and P294S SNPs are located in the SHARPIN UBL domain (Figures 2A, B) (de Rojas et al., 2021; Park et al., 2021; Asanomi et al., 2022). The crystal structure of the human SHARPIN UBL in complex with the human HOIP UBA1-UBA2 revealed that the SHARPIN UBL mainly interacts with the N-terminal helix of HOIP UBA1 (Figure 3D) (Liu et al., 2017). All SNPs reported as AD risk factors on the SHARPIN UBL domain are located on the interaction surface with the N-terminal helix of HOIP UBA1, and may affect the binding of SHARPIN to HOIP. In the crystal structure, the electron density of R274 of SHARPIN was unclear, and its side chain did not form hydrogen bonds or salt bridges with HOIP UBA1. However, a molecular dynamics (MD) simulation suggested that SHARPIN R274 forms hydrogen bonds and salt bridges with E487 and Q490 of HOIP, and the R274W mutation destabilizes the complex at the interface (Park et al., 2021). This simulation was validated by co-immunoprecipitation assays, which showed that the binding between SHARPIN UBL (R274W) and HOIP UBA1-UBA2 was significantly reduced, as compared with that of SHARPIN UBL WT (Park et al., 2021). Furthermore, the R274W mutant of SHARPIN showed aberrant cellular localization and reduced activation of NF-κB (Asanomi et al., 2022). The D291G mutation has also been reported as an AD-related SNP, but the D291 side chain is not directly involved in HOIP binding. The side-chain carboxy group and the main-chain CO group of D291 in SHARPIN hydrogen bond with the main-chain CO group of R220 in SHARPIN and the side-chain amino group of Q481 in HOIP, respectively (Liu et al., 2017). The hydrogen bond between D291 and R220 of SHARPIN may fix the position of D291, but the D291G mutation breaks this hydrogen bond. Furthermore, Gly has a high degree of conformational freedom in the backbone. Therefore, the D291G mutation of SHARPIN would change the conformation and result in the loss of the hydrogen bond between the main-chain CO group of D291 in SHARPIN and the side-chain amino group of Q481 in HOIP. D291 is highly conserved among species, indicating that it may play an essential role in properly folding the UBL domain to bind HOIP (Figure 2B). P294S, another AD-associated SNP, may inhibit the hydrophobic interaction between P294 of SHARPIN and M484 in HOIP. In addition, P294 is located at the N-terminus of β4 and seems to promote the termination of β3 and β4. Therefore, the P294S mutation of SHARPIN may attenuate its hydrophobic interaction with M484 of HOIP and destabilize the SHARPIN UBL folding, thus reducing SHARPIN binding to HOIP.

The NZF domain of SHARPIN preferentially binds M1- and K63- over K48-linked ubiquitin chains (Gerlach et al., 2011; Sato et al., 2011). The ubiquitin chain binding activity of the SHARPIN NZF is indispensable for M1-linked ubiquitination by SHARPIN–HOIP in cells, but not *in vitro* (Ikeda et al., 2011; Tokunaga et al., 2011). A frameshift at L333 is reportedly a risk factor for AD (Figure 2A), and thus the binding of the SHARPIN NZF to the ubiquitin chain may also be required to prevent AD (Asanomi et al., 2022). The structure of the human SHARPIN NZF has not been determined. However, the structure of the mouse HOIL-1L NZF, which shows significant similarity to the SHARPIN NZF (Figure 2C), has been reported as a complex with M1-linked diubiquitin (M1-Ub_2_) (Figures 3E, F) (Sato et al., 2011). The HOIL-1L NZF simultaneously binds the distal and proximal Ub moieties of M1-Ub_2_. Furthermore, the HOIL-1L NZF contains the additional C-terminal helix (NZF tail) that binds to the proximal ubiquitin to enhance the binding affinity for M1-linked chains. Although the SHARPIN NZF does not contain an additional NZF tail, the Arg-Pro sequence is conserved between the NZF domains of HOIL-1L and SHARPIN (R365-P366 of SHARPIN) (Figures 2B, C). Since this dipeptide sequence is a crucial determinant for the M1-linkage-specific binding of the HOIL-1L NZF, the SHARPIN NZF would probably bind to the M1-linked chains by the same mechanisms as the HOIL-1L NZF. AlphaFold2 structural predictions for human SHARPIN (residues 345–387) in complex with M1-Ub_2_ have also highlighted the similarities between the NZF domains of SHARPIN and HOIL-1L (Jumper et al., 2021). The predicted AlphaFold2 model is similar to the crystal structure of the mouse HOIL-1L in complex with M1-Ub_2_ (rmsd value of 1.101, 180 residues total), except for the regions outside the NZF domain of SHARPIN (residues 377–387) (Figure 3G). T358A of SHARPIN is another AD-associated SNP (Asanomi et al., 2022). An aliphatic portion of T201 of mouse HOIL-1L, equivalent to SHARPIN T358, forms a hydrophobic surface to interact with the H68 of the distal ubiquitin (Figure 3F). Furthermore, T201 of mouse HOIL-1L forms a water-mediated hydrogen bond network with the NH groups of A46 and A47 of the distal ubiquitin. Although AlphaFold2 does not predict the positions of water molecules, like T201 of the mouse HOIL-1L, T358 of SHARPIN would bind the distal ubiquitin *via* water-mediated hydrogen bond network in addition to direct contact with the H68 of the distal ubiquitin (Figures 3F, G). Since the T201A mutation of HOIL-1L reduced the affinity for M1-Ub_2_ by 58-fold (Sato et al., 2011), the T358A mutation of SHARPIN would also significantly affect the affinity for the M1-linked chains. In contrast, the effects of W379X and P381R on the SHARPIN NZF could not be predicted. W379 and P381 are located outside the SHARPIN NZF, and while the SHARPIN NZF is well conserved, the C-terminal region outside the SHARPIN NZF is not and appears to be of little importance (Figure 2B). The AlphaFold2 model indicated that this C-terminal region is not involved in binding to the distal and proximal ubiquitin moieties of M1-linked chains. The C-terminal domains of NPLOC4, TAB2, and TAB3 do not contain an extra residue after the NZF domain (Figure 2C). Therefore, this extra region of the SHARPIN NZF may not be required for the folding and functions of the NZF domain.

The effects of AD-associated SNPs in SHARPIN were inferred from the structure, to identify those that attenuate the NF-κB activation by LUBAC. G186R prevents SHARPIN binding to HOIL-1L, while R274W, D291G, and P294S prevent SHARPIN binding to HOIP, thus inhibiting the formation of the LUBAC complex (Figures 3B–D). In contrast, the L333 frameshift mutation and T358A prevent SHARPIN binding to M1-linked chains (Figures 3E–G). Although this interaction does not affect the activity of LUBAC *in vitro*, it inhibits M1-linked chain assembly and NF-κB activation by LUBAC in the cell. However, the effects of the S17F, W379X, and P381R mutations on SHARPIN cannot be predicted, because residues 1–19 and 377–387 are poorly conserved and within flexible loop regions (Figure 2B, Figures 3A, G). Since previous studies confirmed that these regions do not affect the LUBAC complex formation and the M1-linked chain assembly activity by LUBAC *in vitro* (Gerlach et al., 2011; Ikeda et al., 2011; Tokunaga et al., 2011; Fujita et al., 2018), the effects of these mutations on SHARPIN functions in cells must be elucidated.

### 3.3 ALS and heterologous ubiquitination

**FIGURE 4:**
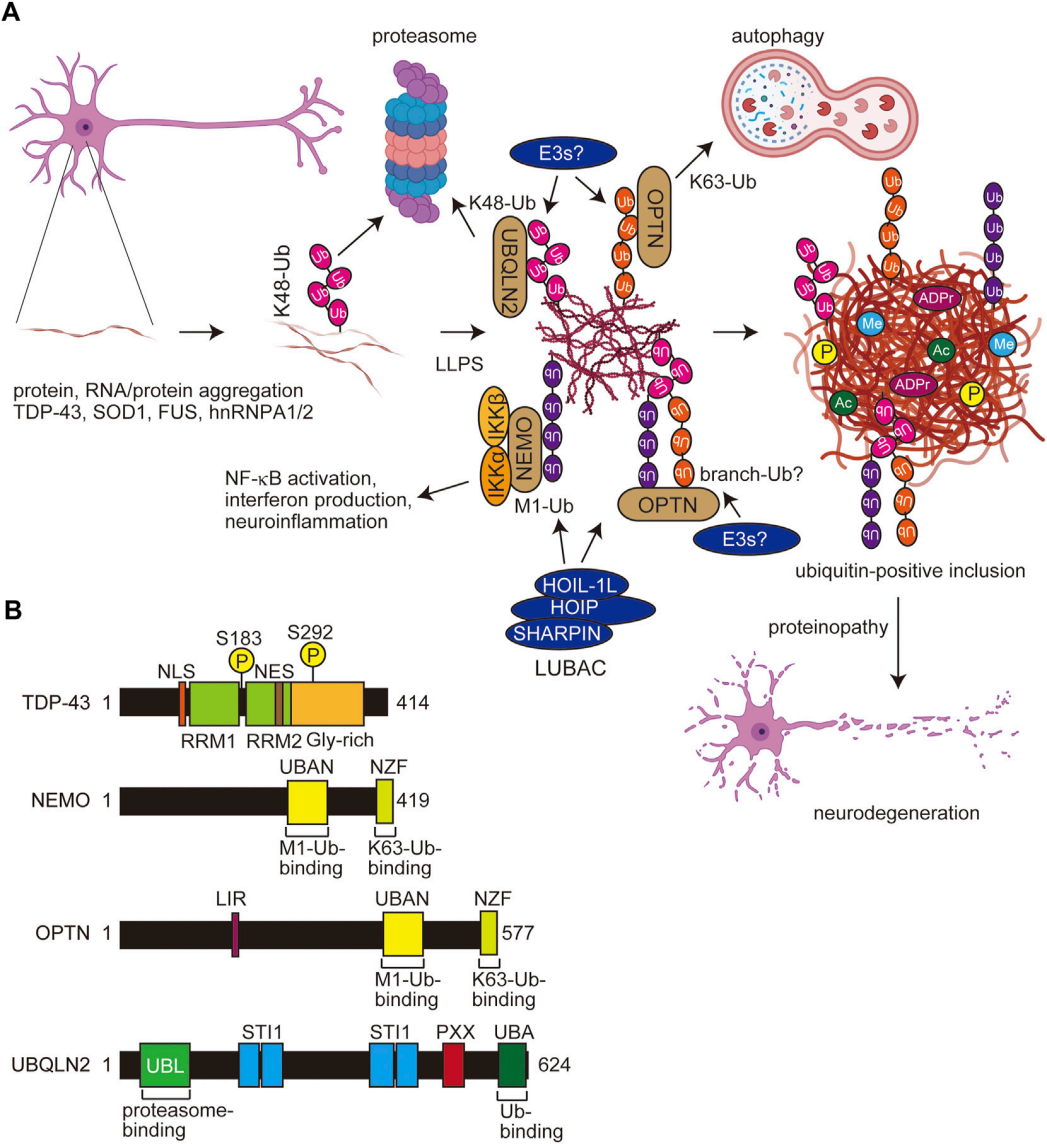
Scheme for PTM-mediated aggregate formation in ALS and domain structures of related molecules. **(A)** In ALS, the misfolding of aggregating proteins such as TDP-43, SOD1, FUS, and hnRNPA1/2 forms thin inclusions, and various PTMs including multiple ubiquitinations facilitate the LLPS and expansion of aggregates. Several E3s, including LUBAC and ubiquitin-binding proteins such as UBQLN2, OPTN, and NEMO, affect the proteasomal and autophagic degradation and the inflammatory response. Finally, the accumulated protein aggregates exert proteinopathy leading to cell death. Ub, ubiquitin; P, phosphorylation; Me, methylation; ADPr, ADP-ribosylation. **(B)** Domain structures of TDP-43, NEMO, OPTN, and UBQLIN2. NLS, nuclear localization signal; RRM, RNA recognition motif; NES, nuclear export signal; UBAN, ubiquitin binding in ABIN and NEMO; NZF, Npl4-type zinc finger; LIR, LC3-interacting region; UBL, ubiquitin-like; STI1, stress-induced protein 1; PXX, Pro-rich repeat; UBA, ubiquitin-associated.

ALS is a fatal neurodegenerative disorder that causes the progressive loss of motor neurons (Taylor et al., 2016; Mejzini et al., 2019). Although most of ALS patients are sporadic (sALS) of unknown onset, ∼10% of ALS cases are familial (fALS), and over 30 potential ALS genes, such as *TARDBP* (which encodes TDP-43), *SOD1*, *C9ORF72*, *OPTN*, *UBQLN2*, and so on, have been identified (Cirulli et al., 2015). These gene products regulate various cellular functions, including RNA metabolism, protein trafficking, proteostasis, protein aggregation, ubiquitin-proteasome system, autophagy, and inflammation (Figure 4A). A genetic mutation in Cu-Zn superoxide dismutase 1 (*SOD1*) was initially identified as a causative gene in fALS (Rosen et al., 1993), and *C9ORF72* is the most common cause of fALS, in which the expansion of a GGGGCC repeat translates into poly-GA, poly-GP, poly-GR, poly-PA, and poly-PR (DeJesus-Hernandez et al., 2011; Renton et al., 2011). The ubiquitinated insoluble cytoplasmic inclusions of TDP-43 are found in 97% of ALS and 45% of FTD cases, indicating that TDP-43 inclusions are a hallmark of ALS/FTD-mediated proteinopathy (Ling et al., 2013). The N-terminal region of TDP-43 contains two RNA recognition motifs (RRM1 and RRM2), which bind RNA/DNA, and a Gly-rich low complexity region at the C-terminus (CTFs) (Figure 4B) (Prasad et al., 2019). TDP-43 also contains a nuclear localization signal (NLS) and a nuclear export signal (NES), suggesting its function as a nuclear-cytoplasm shuttling protein, although native TDP-43 is predominantly localized in the nucleus. TDP-43 is susceptible to several PTMs, such as proteolytic cleavage, hyperphosphorylation, ubiquitination, and neddylation. Proteolysis resulting in the loss of the NLS or mutations in TDP-43, which are mainly found in the CTFs, facilitate the cytoplasmic accumulation of insoluble inclusions with cross-β amyloid structures (Berning and Walker 2019; Arseni et al., 2022; Shenouda et al., 2022). The TDP-43 aggregates reportedly included both the K48- and K63-linked ubiquitin chains, which function in proteasomal and autophagic degradation, respectively (Scotter et al., 2014). Moreover, the age-dependent increase in the K63-ubiquitination of the CTFs of TDP-43 was identified (Yin et al., 2021). The E2 of UBE2E3 (UbcH9), E3s such as parkin, VHL/Cul2, Znf179 (RNF112), Praja1, RNF220, and SCF^cyclin F^, and DUBs such as USP5, USP7, USP8, USP10, USP13, USP14, and CYLD, are reportedly involved in the ubiquitination and deubiquitination of TDP-43 (Tran and Lee 2022), indicating that many E3s and DUBs regulate the spatiotemporal ubiquitin dynamics of TDP-43 in ALS.

Optineurin (OPTN) shares significant sequence similarity with that of NEMO (Figure 4B). We reported that the OPTN UBAN domain selectively binds to M1-ubiquitin in a similar manner to that by NEMO (Nakazawa et al., 2016). Importantly, the fALS-associated *OPTN* mutations in the UBAN domain, such as E478G and Q398X, abolished the inhibitory effects of OPTN on canonical NF-κB activation, and M1-ubiquitin is colocalized with TDP-43 inclusions in neurons from *OPTN*-associated ALS patients (Maruyama et al., 2010; Nakazawa et al., 2016). Although K48-ubiquitin was detectable in tiny TDP-43 inclusions, K63- and M1-positive inclusions were observed in K48-positive thick TDP-43 inclusions (Nakayama et al., 2020). Therefore, the ubiquitin chains apparently become more complex as the ALS disease progresses (Figure 4A). Furthermore, we recently showed that cytoplasmic aggregates of the ectopically expressed, truncated fALS-associated Ala315→Thr (A315T) mutant of TDP-43 in Neuro2a cells are colocalized with M1-, K48-, and K63-ubiquitins (Zhang et al., 2022). These results suggested that TDP-43 inclusions contain various types of ubiquitin chains, and may form complex ubiquitin structures such as branched and/or hybrid chains including M1-chain.

### 3.4 Multiple ubiquitinations in other neurodegenerative diseases and aggrephagy

Not only tau and TDP-43, but M1-ubiquitin is reportedly colocalized with protein aggregates formed by the overexpression of HD-derived polyglutamine proteins and Machado–Joseph disease-associated ataxin-3 (van Well et al., 2019). Importantly, Winklhofer’s group showed that LUBAC is recruited to aggregates of huntingtin-derived polyglutamine (Htt-polyQ), and M1-ubiquitin is co-localized with the aggregates. Furthermore, they indicated that M1-ubiquitin is involved in various disease-associated aggregable proteins. Thus, LUBAC-mediated M1-ubiquitination seems to be crucial as a regulator of multiple neurodegenerative diseases.

In addition to proteasomal degradation, protein ubiquitination also plays an important role in aggrephagy, the adaptor-mediated autophagy of aggregated proteins. In this pathway, ubiquitinated protein aggregates are recognized by autophagy receptors such as p62 (SQSTM1), NBR1, TAX1BP1, NDP52 (CALCOCO2), and OPTN, through the ubiquitin-binding domains. These receptors further involve the LC3-interacting region (LIR) domains, and therefore bridge ubiquitinated cargo and lipidated LC3 for selective autophagy (Vargas et al., in press). In neurodegenerative diseases, hyperphosphorylated tau fibrils (Jo et al., 2014), Aβ (Pickford et al., 2008), Htt-polyQ (Pickford et al., 2008), α-synuclein (Winslow et al., 2010), and TDP-43 (Buchan et al., 2013; Scotter et al., 2014) are aggrephagy substrates. Therefore, the complex ubiquitination of aggregating proteins is likely to affect selective autophagy as well.

## 4 Heterologous ubiquitination and LLPS

During inclusion body formation in neurodegenerative diseases, proteins with low-complexity domains are sorted by LLPS into protein/RNA-rich droplets and membrane-less organelles. Various PTMs, such as phosphorylation, ubiquitination, acetylation, arginine methylation, arginine citrullination, and PARylation, regulate LLPS (Luo et al., 2021). Heterologous ubiquitination and its binding decoders are also involved in LLPS. Importantly, M1-ubiquitin reportedly facilitates fibrillar aggregates formation than that of K48- and K63-linked ubiquitin chains (Morimoto et al., 2015), suggesting that M1-ubiquitination is crucial for the development of neurodegenerative diseases through the promotion of LLPS, oligomerization, and aggregate formation.

The proteasomal shuttle factor ubiquilin 2 (UBQLN2) contains the N-terminal ubiquitin-like (UBL) and C-terminal UBA domains, and mutations in the Pro-rich (PXX) region of *UBQLN2* cause dominant X-linked ALS and ALS/dementia (Figure 4B) (Deng et al., 2011). Ubiquitin binds the UBA domain of UBQLN2, which eliminates LLPS and enables the trafficking of ubiquitinated substrates from stress granules or other membrane-less organelles to protein quality systems (Dao et al., 2018). Importantly, ALS-associated mutations in *UBQLN2* increase oligomerization, and the ubiquitin-binding generally disrupts LLPS, droplets, and aggregates (Dao et al., 2019; Zheng et al., 2021). The effects of polyubiquitin chains on UBQLN2 LLPS are highly dependent on the linkage-types; thus, K11- and K48-ubiquitin chains inhibit LLPS, whereas K63- and M1-ubiquitin chains, which are extended and flexible, significantly enhance UBQLN2 LLPS (Dao et al., 2022). In addition to UBQLN2, K63- and M1-chains also stabilize the LLPS of p62, an UBA-containing protein (Sun et al., 2018; Zaffagnini et al., 2018).

Recently, Du *et al.* reported that M1-and K63-ubiquitin chains induce the LLPS of NEMO, and both the UBAN and the C-terminal Npl4-type zinc finger (NZF) domains, which bind M1- and K63-ubiquitin chains, respectively, are crucial for the LLPS (Figure 4B) (Du et al., 2022). Thus, bifunctional ubiquitin-binding sites are necessary for efficient LLPS. Furthermore, they showed that activated IKK is present within the NEMO condensates formed upon inflammatory stimulation, indicating that the LLPS of NEMO plays an important role in NF-κB activation. Interestingly, M1-linked ubiquitin induces more potent LLPS than that of the K63-chain, and other Lys-linked ubiquitin chains, including the K48-chain, had little LLPS activity (Du et al., 2022). Like NEMO, OPTN has UBAN and NZF domains, and thus it is very easy to speculate that OPTN also causes LLPS in M1- and K63-ubiquitin chain-dependent manners.

## 5 Therapeutic targets for AD and ALS

### 5.1 AD treatment

At present, acetylcholinesterase inhibitors (AChEIs) such as tacrine (tetrahydroaminoacridine), donepezil, rivastigmine, and galantamine are used for the symptomatic treatment of AD (Breijyeh and Karaman 2020). Memantine has been approved as an *N*-methyl d-aspartate (NMDA) antagonist, and monoclonal antibodies such as Aducanumab, Gantenerumab, and so on, which target and remove β-amyloid, are expected to be effective disease-modifying therapeutics (Breijyeh and Karaman 2020).

### 5.2 PROTACs for AD treatment

**FIGURE 5:**
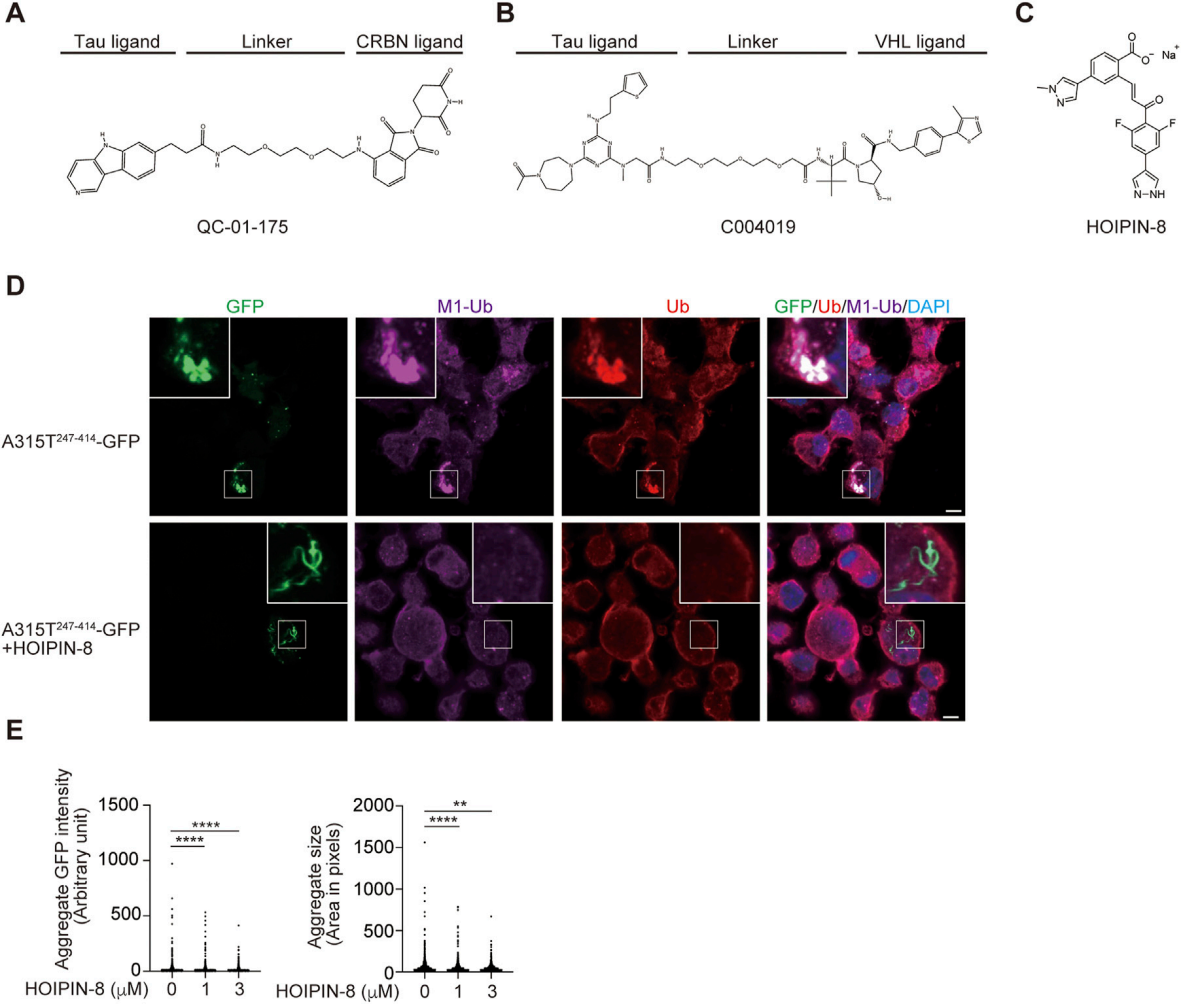
Tau-targeting PROTACs and the LUBAC inhibitor, HOIPIN-8. Chemical structures of the tau-targeting PROTACs QC-01–175 **(A)** (Silva et al., 2019) and C004019 **(B)** (Wang et al., 2021), and the LUBAC inhibitor HOIPIN-8 **(C)** (Katsuya et al., 2019; Oikawa et al., 2020b) are shown. **(D)** Reduced aggregation of truncated TDP-43 in HOIPIN-8-treated cells. The ALS-associated A315T mutant of truncated TDP-43 (A315T^247-414^-GFP) was expressed in Neuro2a cells in the absence or presence of 10 μM HOIPIN-8 for 20 h, and then immunofluorescent staining was performed with GFP or the indicated antibodies (cited from (Zhang et al., 2022). *Bars* = 5 μm. **(E)** The intensities and sizes of the A315T^247-414^-GFP aggregates were reduced by HOIPIN-8. **: *p* < 0.01; ****: *p* < 0.0001.

PROTACs are heterobifunctional molecules that link a protein of interest (POI) and E3s such as CRL4-Cereblon (CRBN), VHL, IAPs, and MDM2, with an optimal linker, leading to the proteasomal degradation of target proteins (Sakamoto et al., 2001; Bekes et al., 2022). PROTACs are highly anticipated as next-generation therapeutic drugs, and several compounds targeting androgen receptor (AR), estrogen receptor (ER), Bcl-xL, IRAK4, BTK, *etc.* are in clinical studies (Bekes et al., 2022). At present, more than 3,700 PROTACs have been reported (Weng et al., 2023). To treat neurodegenerative diseases, PROTACs, which target tau, α-synuclein, mHTT, TDP-43, and FUS, are being developed. For the treatment of tauopathy in AD and FTD, peptide-based PROTACs for tau such as TH006, which contains a tau recognition sequence (YQQYQDATADEQG), short linker, VHL recruit motif, and cell-penetrating poly-Arg region, were initially constructed and decreased the tau levels in the cells and the brains of an AD mouse model (Chu et al., 2016). Similarly, peptide-directed PROTACs that link the tau recognition sequence and the Keap1-Cul3 ligase induced the intracellular degradation of tau (Lu et al., 2018). Importantly, Silva *et al.* developed hetero-bifunctional molecules, such as QC-01–175, which links the PET probe-derived tau ligand of T807 and the CRBN ligand, pomalidomide, that ameliorate tauopathy in FTD-patient derived neuronal cells (Figure 5A) (Silva et al., 2019; Silva et al., 2022). Furthermore, Wang *et al.* developed the PROTAC named C004019, which links a tau ligand and a VHL-CUL2 binder and promotes the degradation of overexpressed human tau in HEK293 and SH-SY5Y cells, and decreases the tau levels in the brains of wild-type and tau-transgenic mice (Figure 5B) (Wang et al., 2021).

### 5.3 ALS treatment

Currently, riluzole (a glutamic neurotransmission inhibitor) and edaravone (an antioxidant) are used for ALS treatment, although their efficacies are limited (Jaiswal 2019). Therefore, there is an urgent need to develop new therapeutic agents. Several protein kinase inhibitors, such as a c-KIT receptor inhibitor (Masitinib), a ROCK inhibitor (Fasudil), a Src/c-Abl inhibitor (Bosutinib), an mTOR inhibitor (Rapamycin), a RIPK1 inhibitor (DNL747), sodium phenylbutyrate-taurursodiol (Paganoni et al., 2020), and others are currently in clinical trials for ALS patients, and many protein kinase inhibitors are undergoing pharmaceutical development (Palomo et al., 2021). Furthermore, antisense oligonucleotides (ASOs), RNA interference, and antibody-based methods are conducted (Amado and Davidson 2021), and the administration of ASOs targeting ataxin-2 reduced the TDP-43-positive inclusions and prolong survival (Becker et al., 2017).

### 5.4 LUBAC inhibitor, HOIPIN-8

We screened 250,000 small molecular chemicals, and identified a thiol-reactive, α,β-unsaturated carbonyl-containing chemical compound, named HOIPIN-1 from HOIP inhibitor-1, as a LUBAC inhibitor (Katsuya et al., 2018). We developed derivatives of HOIPIN-1, and found that HOIPIN-8 is the most potent LUBAC inhibitor among them (Figure 5C) (Katsuya et al., 2019). HOIPINs are conjugated to the active site Cys885 in HOIP through Michael addition, and inhibit the RING-HECT-hybrid reaction (Oikawa et al., 2020b). Importantly, HOIPIN-8 further masks the critical residues for acceptor ubiquitin-binding residues in the LDD of HOIP (Figure 1). HOIPINs suppress the higher intracellular linear ubiquitin levels upon stimulation with inflammatory cytokines, and attenuate the enhanced NF-κB activity in *OPTN*-deficient cells (Oikawa et al., 2020b). We found that the genetic ablation of *Hoip* or the treatment with the HOIPIN-8 reduced the aggregates of the ALS-associated A315T mutant of truncated TDP-43 in Neuro2a cells (Figure 5D), and the quantitative analysis revealed that the intensities and sizes of the aggregates were significantly reduced by HOIPIN-8 (Figure 5E) (Zhang et al., 2022). These results suggest that LUBAC is a novel regulator of TDP-43 proteinopathy in ALS, and LUBAC inhibitors may be effective as disease-modifying agents for ALS. Accordingly, *in vivo* drug efficacy analyses using ALS model mice are necessary.

## 6 Perspectives and impact

In this review, we have summarized the findings detailing how various types of ubiquitination and deubiquitination, including M1-ubiquitin chains, are involved in aggregate formation and proteinopathy in AD and ALS. Ubiquitination causes changes in the physical properties and cell functions of aggregable proteins, and is deeply involved in the progression of neurodegenerative diseases. It is likely that the involvement of non-Lys ubiquitination and non-protein ubiquitination in these diseases will also be revealed. The development of E3 inhibitors functioning in ubiquitin-positive aggregate formation could suppress neurodegenerative diseases, and since M1-ubiquitin can only be produced by LUBAC, it is an important therapeutic target. PROTACs may also become effective tools in the future. If E3 proteins such as RNF182 and TRIM9, which are specifically expressed in the central nervous system, can be linked to target proteins, then it may be possible to develop therapeutic drugs for neurodegenerative diseases with minimal side effects (Bekes et al., 2022). Furthermore, the development of aggregate protein-specific AUTACs (autophagy-targeting chimeras) (Takahashi et al., 2019), which lead target proteins to autophagy, may also be an effective therapeutic strategy. Neurodegenerative diseases are intractable diseases that currently lack effective treatments, and we believe that it is extremely important to pursue all possibilities aimed at developing treatment methods, including ubiquitination.
